# Editorial: Insights into glyco-parasitology

**DOI:** 10.3389/fmolb.2024.1422955

**Published:** 2024-05-10

**Authors:** Kentaro Kato, Jamie Heimburg-Molinaro

**Affiliations:** ^1^ Department of Eco-epidemiology, Institute of Tropical Medicine (NEKKEN), Nagasaki University, Nagasaki, Japan; ^2^ School of Tropical Medicine and Global Health, Nagasaki University, Nagasaki, Japan; ^3^ National Center for Functional Glycomics, Department of Surgery, Beth Israel Deaconess Medical Center and Harvard Medical School, Boston, MA, United States

**Keywords:** parasites, helminths, protozoa, glycans, lectins, glycosylation

“Glycoscience” and “Parasitology” are complex, long-studied fields of science, with enormous efforts focused on both scientific areas. The combination of “Glyco-parasitology” brings together the important and unique features of glycans related to parasites, parasite biology and immune functions, with many questions still unexplored. This is partially because life cycles, generation of glycans, and functions of glyco-related molecules of parasites and hosts themselves are complicated and yet to be fully defined. For example, expression of glycoforms (glycan structures) differ among each life stage of a parasite and susceptibilities to parasitic infection differ among the strains or individuals in the host species. These features may create difficulties in merging Glycoscience and Parasitology, but there are cutting-edge studies happening across the world and we have brought together a collection of them for this Research Topic, “Insights into Glyco-parasitology”. 

It is not a stretch to say that parasitic infections are mediated by glyco-related molecules. Many of the parasites utilize their glyco-binding molecules, some of which may be glycosylphosphatidylinositol (GPI)-anchored, to attach to and infect hosts (Loukas and Maizels, 2000; Petri et al., 2002; Harcus et al., 2009). Some transfer host glycans to themselves to evade the host’s immune responses (Campetella et al., 2020). In some cases, glycan-glycan interactions are important to determine and drive parasite-host interactions (Hall et al., 2020). On the host side, many glycans and glyco-related molecules of parasites are antigenic to humans, and cause or modulate an immune response. In this Research Topic, Prasanphanich et al. showed that IgG antibodies, generated during infection, targeted *Schistosoma mansoni* complex-type *N*-glycans with core α2-xylose and core α3-fucose. These antibodies could kill schistosomula in a complement-dependent manner. From the results, we can see an exciting possibility for vaccine development targeting the core xylose/core fucose glycans, and such vaccines are not yet available towards *S. mansoni* (Bunte et al., 2022).

Some immune cells attach to and phagocytose the parasites via glycan interactions, and thereby, the parasites are able to infect and proliferate in the immune cells (Tanaka et al., 2007; Lefèvre et al., 2013). Membranous surfaces are covered by mucous consisting mainly of mucins and other glycoproteins, which are used for maintaining the humidity of the body, protecting from physical damage, retaining beneficial microorganisms and preventing virulent microorganisms from entering (Garić et al., 2020; Paone and Cani, 2020). Mucins are components of tears, saliva and the mucous layers covering digestive and respiratory tracts. Cells are also covered by glycomolecules-the glycocalyx. These *N*- and *O*-glycans on mucins, glycoproteins and glycolipids often function as the attachment and entry point for parasites. In this case, parasite glycan-binding proteins (GBPs) are important for infection. Some of the GBPs serve as virulence factors with their hemagglutinating, hemolytic and cytotoxic activities (Singh et al., 2016). If there is a mechanism to prevent the attachment of parasitic GBPs to glycans and glyco-molecules, or develop antibodies against carbohydrate recognition domains (CRDs) of the lectins, the parasites will be stifled in attaching to host cells, decreasing the impact of disease in humans.

As mentioned above, many of the parasitic glycans are different from human structures, so our immune systems can recognize those as foreign antigens and eliminate the parasites from our body. These glycans can be utilized as diagnostic tools and vaccine targets. Parasitic glycan-based vaccination may be effective in protecting from infection, or lessening the symptoms from infection. However, the story is not that simple. Some types of parasitic glycans can suppress the host immune response, and thereby, the parasites can persist in the hosts for many years (van Die and Cummings, 2010). *Trypanosoma* spp. utilize their trans-sialidases to transfer host sialic acids onto parasitic glycans to hide their antigenicity and escape from host immune recognition, though attempts to generate vaccines targeting these molecules have been made (Silva et al., 2009; da Costa et al., 2021). In this aspect, it is important to clarify which parasitic glycans suppress the host immune response and which parasitic glycosyltransferases/glycosidases relate to virulence of the parasites. So far, very few studies have been conducted to identify such glycans and glycosyltransferases of parasites, although some glycomics data are available (Hokke et al., 2007) and glycan synthetic pathways can be drawn (Izquierdo, 2023). In this Research Topic, Wang et al. reviewed *N*- and *O*-linked glycan and GPI-anchor synthetic pathways of *Cryptosporidium* parasites by reanalyzing the *Cryptosporidium* genomes. The authors assembled elegant diagrams of glycosylation-related pathways as a comprehensive resource for the glyco-parasitology research communities.

Another mode of action of parasitic glycans, glycoproteins and glycolipids is conferred by secretion of molecules from the parasites. The glyco-related molecules on extracellular vesicles (EVs) including exosomes may also affect host cells and environments. Interestingly, *S. mansoni* EVs contain numerous glycan structures, including sialylated glycoconjugates that seem to be obtained from exogenous sources because the parasite cannot synthesize sialic acids (Dagenais et al., 2022). Kuipers et al. showed life stage-specific glycosylation of EVs from *S. mansoni* and those glycans may have an affect on immune cells through interactions with specific C-type lectins on the host cells, including MGL and DC-Sign. This research field is relatively new and we are expecting exciting advancements in the future.

Finally, biosynthetic and metabolic pathways of parasites can be targets for drug development, if the pathways are different from those of hosts. For example, some parasites solely depend on their energy synthesis from the glycolysis pathway. In this case, if we can find or develop competitors that bind to glucose transporters and receptors, these molecules may be used as preventive or therapeutic drugs. One of these candidates would be rare sugars, as described by Harada et al., such as D-allose and D-psicose (Harada et al., 2012). Kato et al. showed that D-allose and D-psicose have potential as amoebicides by inhibiting proliferative steps, however additional information on the characteristics of the sugar transporters and receptors of the parasites is needed to exploit these findings.

In summary, this Research Topic “Insights into Glyco-parasitology” is a collection of recent research articles, the results of which will certainly serve as the basis of future Glyco-parasitological research. We still have a vast field to explore to clarify how the parasites utilize glyco-related molecules to co-evolve and co-exist with their hosts, and subvert the host defensive strategies. These molecules may have diverse functions among different parasite species, but research targeting these glyco-molecules may lead to new strategies for preventing and treating parasitic infections.
